# Report of a Joint Cancer Research UK/Medical Research Council workshop on cancer cachexia research at the Royal College of Physicians, Tuesday, 2 December 2003

**DOI:** 10.1038/sj.bjc.6602565

**Published:** 2005-04-19

**Authors:** T E Steer

**Affiliations:** 1 Medical Research Council, Resource Centre for Human Nutrition Research, Elsie Widdowson Laboratories, Fulbourn Road, Cambridge, CB1 9NL, 01223 426356, UK

**Keywords:** cachexia, Cancer Research UK

## Abstract

A joint workshop held by Cancer Research UK and the Medical Research Council aimed to stimulate interest in further research into the area of cancer cachexia. The workshop was divided into four sessions: an overview of cancer cachexia, potential mechanisms involved and methodologies that might be used to understand cachexia, and also the experience of cachexia from other disease areas. The workshop identified a need to develop a multimodal therapeutic approach to cancer cachexia and a need to undertake more multidisciplinary research.

## BACKGROUND TO THE WORKSHOP

In 2002, Cancer Research UK (CR UK) undertook a strategic review of issues facing cancer research. The review highlighted a lack of research activity and understanding of the physiological mechanisms of whole-body responses to the presence of a tumour including cancer cachexia. Following discussions with the Medical Research Council (MRC), both funders agreed to hold a joint workshop on cancer cachexia as an initial step in raising the profile and stimulating high-quality research in this area. The aims of the Workshop would be to introduce issues in cancer cachexia research to a wider UK clinical oncology research audience, and to highlight research methodologies that might be used to investigate cachexia in cancer patients and to draw on the experiences and progress of cachexia research in other disease areas.

## PRESENTATIONS

The Workshop presentations were divided into four sessions: an overview, a review of the potential mechanisms involved in cancer cachexia, methodologies that might be used to assess and understand cachexia, and the experience and knowledge of researchers from other disease areas.

## OVERVIEW

Professor Robert Souhami of CR UK introduced the workshop by saying that an understanding of the underlying mechanisms was a prerequisite to the development of therapeutic interventions in cachexia. For this reason, the workshop would explore possibilities for UK collaborative research embracing oncologists, scientists with expertise in intermediary metabolism and acute-phase response (APR), and also scientists with relevant specific technological expertise and those in the field of biochemistry and physiology.

### Cancer cachexia: mechanisms and clinical implications

Professor Kent Lundholm (Göteborg, Sweden) described how resting energy expenditure (REE) was significantly increased compared to controls (patients with chronic inflammation who experienced some weight loss). Fundamental changes in metabolism, especially fat oxidation, appear to be related to the anorexia and stress response that cachectic patients experience. The likely explanation for weight loss was depressed protein synthesis, rather than increased protein breakdown. He suggested that the weight loss experienced by cancer cachexia patients was not caused primarily by a decrease in food intake (anorexia) in all patients, and suggested that patients may need to decrease their physical activity to preserve body mass (Boaseus *et al*, 2001).

In discussion, the question was raised as to how a reduction in physical activity might help attenuate weight loss in cancer cachexia patients, since a reduction in physical activity would result in reduced lean body mass (LBM). While Professor Lundholm agreed that this was true, he pointed out that if cancer patients do not compensate by increased eating then physical activity would accelerate weight loss.

## POTENTIAL MECHANISMS OF CANCER CACHEXIA

### Molecular mechanisms of cancer cachexia

Professor Josep Argilés (University of Barcelona, Spain) described the anorexia-cachexia syndrome as being characterised by marked weight loss, anorexia, asthenia and anaemia, leading to a malnourished state (Argiles *et al*, 2003). This was caused by the induction of anorexia and an accelerated catabolic state which in turn promotes severe metabolic disturbances in the host, including hypermetabolism, associated with increased energetic inefficiency and increased thermogenesis. A tumour has a high demand for glucose which is supplied via breakdown of adipose and lean tissue. The breakdown of adipose tissue may also release cytokines, growth factors and acute-phase proteins which may also influence metabolism.

He concluded that a better understanding of the role of cytokines, which interfered with the molecular mechanisms accounting for protein wasting in skeletal muscle, was essential for the design of future effective therapeutic strategies.

### Biochemistry mechanism of tissue catabolism

Professor Mike Tisdale (Aston University) suggested that in cachectic patients lipid-mobilising factor (LMF) may stimulate uncoupling proteins which may utilise excess lipid that has been mobilised during fat catabolism.

Proteolysis-inducing factor (PIF) causes catabolism of skeletal muscle. Proteolysis-inducing factor initiates intracellular protein degradation in skeletal muscle and inhibits protein synthesis (Whitehouse and Tisdale, 2003). Both LMF and PIF could be described as tumour catabolic factors and may play a role in cancer cachexia.

### Cancer cachexia and the APR

Professor Ken Fearon (Edinburgh University) confirmed that pro-inflammatory cytokines such as IL1 and IL6 have long been established as playing a major role in experimental models of the cachexia syndrome. The APR represents a marker of proinflammatory cytokine activity *in vivo* and may also be a mechanism contributing to weight loss in cancer. Recent evidence links the presence of an APR with both the anorexia and hypermetabolism in cachectic patients (Wigmore *et al*, 1997).

Professor Fearon suggested that systemic inflammation had been shown to be an adverse risk factor for survival in advanced cancer and was, therefore, a valid target as part of a multimodal approach to the treatment of cancer. However, it was not clear what initiated systemic inflammation in cancer.

### Grehlin: a promising approach

Professor Bloom (ICSM, Hammersmith Hospital) stated that ghrelin is a hormone secreted from the gastric cells that stimulates food intake in healthy volunteers at physiological concentrations. He had investigated whether the effect of intravenous ghrelin in cancer patients with appetite loss could be an effective treatment. Seven anorectic patients attended for a ghrelin (5 pmol kg^−1^ min^−1^) infusion and a saline control in a randomised, double-blinded study. Food intake from a buffet meal was measured after 90 min of infusion. Patients rated the pleasantness of the meal on a visual analogue scale. Energy intake from the buffet meal was increased by 31±7% (*P*=0.02) on the ghrelin infusion day, when every patient ate more, compared with saline infusion. Patients also found the meal more pleasant on their ghrelin day (23% increase of visual analogue score ±8%; *P*=0.02). This large increase in energy intake suggests that regular ghrelin treatment could be an effective treatment for patients with loss of appetite (Neary *et al*, 2004).

### General discussion

In the general discussion that followed this session, the APR emerged as an area of potential interest. The discussion covered the direct relationship of APR with the cachexia syndrome and to what extent the production of APR is responsible for cachexia. In addition, it was also pointed out that APR is now recognised as an adverse prognostic indicator and that this is independent of stage of disease.

The roles of nutrition support and chemotherapy were also raised. Views on nutrition support in humans appeared mixed with some evidence (from animal models) to suggest that nutrition support decreased survival rates since it provides substrate for the tumour, while others took the view that it had no adverse risks and allowed patients to better tolerate aggressive anticancer therapies. What remains to be proven is that reducing cancer cachexia *per se* increases survival in cancer patients. Therefore, the importance of retaining fat and LBM and reducing muscle wastage to improve quality of life is paramount.

## POTENTIAL METHODOLOGIES THAT MIGHT BE USED TO ASSESS WEIGHT LOSS AND CHANGES IN ENERGY METABOLISM

### Measuring intermediary metabolism

Professor Ian Macdonald (University of Nottingham) outlined a variety of techniques that are available to measure intermediary metabolism in human subjects, ranging from whole body assessments of energy expenditure, to studying local tissue or organ uptake, oxidation and storage of specific metabolites. However, most of the techniques to measure body composition have been validated in normal individuals where body composition is not drastically altered (Jebb, 1995), rather than in cachectic individuals.

Professor Macdonald said that cancer cachexia was associated with substantial alterations in intermediary metabolism, both at the local tissue and the whole-body level. An integrated approach is required to determine the mechanisms underlying the development of cachexia. This would combine whole-body and regional measurements of metabolism and function, evaluating changes in gene expression in tissues such as muscle and adipose tissue, and establishing the role of cytokine and other molecules released from tumours in mediating these alterations in metabolism.

### Potential of stable isotopes: studies of human metabolism

Dr Andy Coward (MRC Human Nutrition Research) described the principles behind the use of stable isotopes. Most elements exist in more than one stable form and the less abundant ones can be used as tracers. Thus, unlike the situation with radioactive isotopes, which now have very limited use in human studies, there are very few metabolic processes that are not amenable to investigation. Limitations are imposed only by measurement technology, the need for resilient models of the system under study and the processes by which model parameters need to be translated into clinically meaningful values. However, to make the best of the current situation, the appropriate fit between clinical need and technological capabilities always has to be found; thus the modelling and translation processes are equally important (Rennie, 1999). Where consensus for these exists, such as in relation to the measurement of body composition by ^2^H_2_O dilution or energy expenditure by ^2^H and ^18^O turnover in body water (doubly labelled water (DLW) method), much progress has been made. The DLW method measures the rate constants of the disappearance of ^18^O and ^2^H from body water, converts these into values for CO_2_ production and thence energy expenditure, but it is pertinent to ask if these conversions, which represent the modelling and translation process, are really necessary when the original turnover values are closest to the basic physiology.

### Potential of magnetic resonance imaging (MRI) and spectroscopy (MRS)

Professor Peter Morris (University of Nottingham) described MRI as a highly developed technique that can examine the function of both the cortex and the deeper grey matter in the brain. The basis of the contrast, and hence clarity, in MRI is essentially the differing levels of water content of each of the tissues, which tends to increase in cancer. In addition, MRI can observe function through detecting changes in blood oxidation. He suggested that changes in LBM and adipose tissue in cancer patients may be measured at specific body regions over time by selecting or suppressing the MR signal from these tissues on the basis of their differing resonant frequencies (Morris, 1999).

In addition to structural MRI, dynamic and functional assessments are also possible, including flow, perfusion, diffusion, cardiac performance, lung ventilation, gastric emptying and functional neuro-imaging. ^1^H MRS enables the concentrations of metabolites occurring at sufficiently high concentration (mM) to be determined in regions of interest or low-resolution spectroscopic imaging studies.

### Metabolic gas exchange measurements in patients with chronic heart failure

Dr Andrew Clark (Hull University) presented a technique used to measure metabolic gas exchange in heart failure cachexia. Cardiac cachexia is common in heart failure patients and loss of lean tissue is often observed early on in the disease. The rate of oxygen consumption and CO_2_ production is determined experimentally from the difference between inspired *vs* expired gas concentration, and, at the same time, ventilation can also be measured. Exercise capacity is reduced in chronic heart failure. This is characterised by a reduction in peak oxygen consumption and an increase in the ventilatory response to exercise, which is represented by an increase in the slope of the relationship between ventilation and carbon dioxide production. Dr Clark concluded by saying that metabolic gas exchange measurements can be made using straightforward, readily available equipment. The results give an insight into energy metabolism at rest, but more particularly into the physiology and pathophysiology of exercise performance (Anker *et al*, 1997).

In the discussion, it emerged that, although there were some striking similarities, no formal comparisons had been made between cardiac cachexia and cancer cachexia.

## LESSONS FROM OTHER AREAS

### Gastro-intestinal inflammation

Professor Ian Sanderson (St Bartholomew's and The Royal London School of Medicine and Dentistry) said that children with Crohn's disease and children with cachexia both exhibited impaired nutritional state, decreased fat and muscle mass. The inflammation in Crohn's disease also resulted in the expression of cytokines, which could affect appetite, growth and energy expenditure.

Professor Sanderson described transgenic techniques that allowed selective alterations of the expression of genes in the intestinal epithelial cell. Thus, genetically engineered epithelial expression of chemokines increased neutrophil recruitment, a feature of Crohn's disease. Furthermore, luminal molecules such as short-chain fatty acids regulated chemokine expression (Sanderson, 2004). This pathway from the lumen to the epithelium was one example of how nutrients could alter intestinal inflammation.

### Chronic heart failure

Dr Stefan Anker (National Heart and Lung Institute, London) introduced this area by suggesting that the greater the body mass index (BMI), the better the prognosis for survival in patients with heart failure. Patients with cardiac cachexia had a poor prognosis, as did those who were simply thin even in the absence of cachexia. A 1% weight loss indicated a 12% increased risk of impaired survival which was independent of BMI (Anker *et al*, 2003).

Dr Anker said that the pathophysiology of cardiac cachexia was complex and involved neuroendocrine activation, hormone resistance and lack of anabolism, and immune activation and inflammation. Use of interventions targeting neuroendocrine activation provided the strongest evidence that the development of cachexia can be prevented by an average of 8 months. He concluded by stating that fat represents energy that is required to maintain function during disease states and the level of fat tissue has been shown to be a positive predictor of survival.

### Energy metabolism with HIV

Dr Derek Macallan (St George's Hospital, London) suggested that many patients with AIDS were dying from their starvation as well as the disease process. However, unlike cardiac cachexia, fat mass correlated very poorly with survival in patients with AIDS. He said that there was a lot of heterogeneity of mechanisms of weight loss in AIDS and this could well apply to cancer since there are many forms of cancer. For example, patients with opportunistic infections lost weight during the infection but regained some following recovery, but patients with chronic diarrhoea lost weight progressively, with no weight gain. In patients with AIDS and pneumonia, REE increased and lean tissue was preferentially depleted as adipose tissue was preserved, but in protozal diarrhoea REE decreased and adipose tissue was utilised (Macallan *et al*, 1995). He suspected that this could also be the case with cancer patients who would also experience a mix of starvation and catabolic responses.

## SUMMARY

It was agreed that investigation of the mechanisms of cachexia in different disease states could provide informative data with which to develop a multimodal therapeutic approach to this important clinical syndrome. There was a need for more multidisciplinary working especially bridging from academic science clinical to oncologists.

Following the Workshop, both MRC and CR UK wished to encourage high-quality research proposals, and, where appropriate, a multidisciplinary approach was to be encouraged.

## Appendix

**List of participants**
